# Aurones: A Golden Resource for Active Compounds

**DOI:** 10.3390/molecules27010002

**Published:** 2021-12-21

**Authors:** Ilaria Mazziotti, Giovanni Petrarolo, Concettina La Motta

**Affiliations:** Department of Pharmacy, University of Pisa, 56126 Pisa, Italy; i.mazziotti@studenti.unipi.it (I.M.); giovanni.petrarolo@phd.unipi.it (G.P.)

**Keywords:** aurones, polyphenols, flavonoids, synthesis, functional activity

## Abstract

Deemed as poorly represented in nature, aurones have been often overlooked by researchers compared to other members of the flavonoid superfamily. However, over the past two decades, they have been reassessed by the scientific community, who are increasingly appreciating their ability to modulate several biological pathways. This review summarizes the recent literature on this class of compounds, which has been analyzed from both a chemical and a functional point of view. Original articles, reviews and editorials featured in Pubmed and Scifinder over the last twenty years have been taken into account to provide the readers with a view of the chemical strategies to obtain them, their functional properties, and their potential of technological use. The resulting comprehensive picture aims at raising the awareness of these natural derivatives as effective drug candidates, fostering the development of novel synthetic analogues.

## 1. Introduction

In the early twentieth century, Gustav Klein coined the term *anthochlor* (*anthos* = flower, *chlōrós* = yellowish) to define a class of water-soluble pigments conferring color to plants able to synthesize them as secondary metabolites [1]. It included a restricted group of derivatives known as aurones (*aurum* = gold), due to the bright yellow/gold color that these compounds give to plants in which they are located.

**Figure 1:**
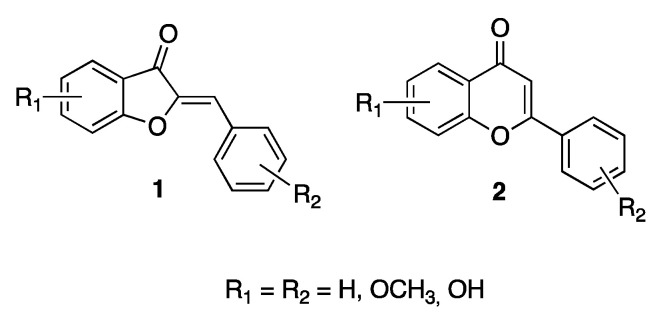
General structures of aurones **1**, and flavones, **2**.

Aurones (**1**, Figure 1) are part of the wide family of polyphenols. More specifically, they may be acknowledged as the lower structural counterparts of the best-known flavones (**2**, Figure 1), a subclass of flavonoids. Actually, as has been observed in detail, the basic structure of an aurone consists of a main 6:5 benzofuranone core, instead of the 6:6 chromane ring of flavones, but shares a 2-aryl decoration with the higher homologues [2].

Deemed as poorly represented in nature, aurones have been often overlooked by researchers compared to the other members of the flavonoid superfamily. However, in recent decades, they have been reassessed and more and more chemists, biologists, and practitioners are taking an interest in their structural and functional peculiarities. Actually, to date, the chemical structures of more than 100 different aurones have been identified, characterized by distinctive hydroxylated, methoxylated, and glycosylated substitution patterns. Moreover, their effective potential to predict viable therapeutic uses has begun to be unveiled [3,4,5,6]. Therefore, they represent a worth deepening class of natural compounds intended to provide bioactive compounds in the near future.

**Figure 2:**
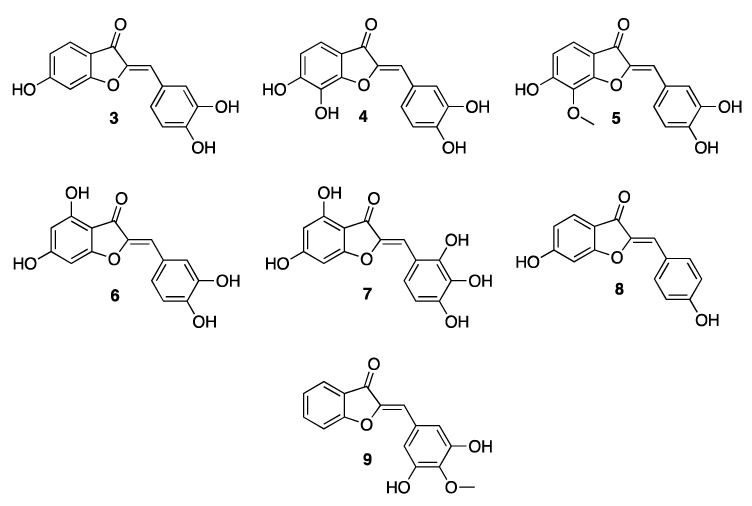
Chemical structures of 4-deoxy- and 4-hydroxy-aurones.

The first examples of aurones were characterized in 1940 in Asteraceae [7], the family of sunflowers, which synthesize the most common 4-deoxy-derivatives of the family including sulfuretin (**3**, Figure 2), maritimetin (**4**, Figure 2), leptosidin (**5**, Figure 2), and their corresponding glycosides. The species *variabilis* and *sulphureus* mainly express sulfuretin (Figure 2) and its glycosylated counterparts in leaves and petals. In the *bidens* species, maritimetin has been isolated while, in the genus Coreopsis, compounds such as sulfuretin, maritimetin, but also leptosidin may be found.

In addition to Asteraceae, aurones are synthesized as the secondary metabolites of many dicotyledons including Anacardiaceae, Cactaceae, Fabaceae, Gesneriaceae, Moraceae, Oxalidaceae, Plumbaginaceae, Rubiaceae, Rhamnaceae, Rosaceae, and Plantaginaceae [4]. Moreover, they have been found in some species of monocotyledons as well as in Bryophytes, as reported by recent studies [8].

While the 4-deoxy-aurones are mainly found in the flowers of the Asteraceae, the 4-hydroxylated derivatives, such as aureusidin (**6**, Figure 2) and bracteatin (**7**, Figure 2), are more common in other plant families, such as in Plantaginaceae, and particularly in the genera Misopates and Linaria, as well as in Rubiaceae and Plumbaginaceae. The flower of the snapdragon plant, defined by the scientific name of *Antirrhinum majus*, is probably the reference natural source of aurones, as high concentrations of the glycosilated form of aureusidin, but also bracteatin, may be found in the vacuoles of the epidermal cells of the petals [9].

Aurones are generally detected in petals or leaves but have also been found in different parts of plants such as leaves, nectar, seeds, wood, and also bark. Hispidol (**8**, Figure 2) and leptosidin, with their glycosylated derivatives, have been found in the seeds of plants such as *Retama raetam* and *Psophocarpus tetragonolobus*, both belonging to the Fabaceae family [4]. Sulfuretin is in charge for the yellow color of the young stems of the deciduous shrub *Cotinus coggygria*, belonging to the Anacardiaceae family, so much so that since ancient times it has been extracted as a pigment to be used as a textile dye [10].

The presence of aurones in primitive bodies such as bryophytes suggests their importance for the vital economy of plants; they pass on the genetic information for the synthesis of these compounds from generation to generation, preserving their expression. In addition to an undoubted natural relevance, due to their role as dyes, aurones are also emerging as possible functional agents, being able to modulate the activity of several biological pathways. Accordingly, they represent an intriguing reservoir of compounds waiting to be fully investigated and used by the scientific community.

## 2. Aurones as Natural Dyes

The color of flowers is the keystone of the evolutionary process that marked the transition from a random pollination, mediated exclusively by the wind, to the use of a vector, that is, insects, birds, and lizards. Being attracted from the nectar of flowers, vectors confer an advantage to the plant world, which may benefit from a greater chance of survival thanks to the passage of pollen from one plant to another and from one place to another. This is why, over the course of time, flowers and plants in general have equipped themselves to attract more and more vectors, in particular by expressing pigments that match the perceptions of these animals [11].

When walking in nature, the characteristic that most catches the eye is a bright yellow color. This is due to aurones and turns out to be more attractive than other shades of yellow given by other parent compounds such as flavonols. Actually, overall, aurones can be acknowledged as the brightest polyphenol pigments in the yellow color range, like anthocyanins are for the red/purple spectrum. The visual contrast that is perceived is the result of different UV absorption spectra. Flavones, isoflavones, and flavanones exhibit UV absorption at 350 nm and, for this reason, they are not characterized by any visible color. Instead, aurones have an absorption spectrum in the 390–430 nm range, thus resulting in a more intense colors than the parent chalcones, showing UV absorption in the 365–390 nm range, and flavonols, having 350–390 nm as the reference range [12]. 

Being characterized by a brilliant color, aurones play a key role in pollination, attracting vectors towards the flowers and to the pollen. Their distribution in the petals of flowers is generally unique. For example, in the case of *A. majus*, the production of aurones is limited to the upper side of the petal, but also affects two additional stripes at the level of the throat, mainly surrounded by a magenta or pink color due to the presence of anthocyanins. The brilliant stripes have the crucial role of warning the insect on the place of nectar [13]. There is also the case where the flowers appear completely yellow, but they this is not so: the contrast is perceived as two-tone by UV-sensitive insects, and these different colors act as a guide for nectar, as was observed in *Bidens ferulifolia* and *Coreopsis gigantea*. This is an example of the evolutionary adaptation of flowers, to better attract pollinating insects such as bees [14]. 

However, as ever, there is always the exception that proves the rule: a particular aurone does exist, 3′,5′-dihydroxy-4′-methoxyaurone (**9**, Figure 2), inducing a red/scarlet color in the nectar of the flowers of specific plants such as the Mauritian *Nesocodon mauritianus*. This is an almost unique exception as this compound provides a color that is not bright yellow. However, the special shade is thought to be due to the particularly alkaline pH of the nectar, which causes the deprotonation of the hydroxyl group of the aurone, leading to a different electronic delocalization [15].

From a commercial point of view, the color of the flowers is of great interest. In this regard, the use of aurones has been thought of to create transgenic plants endowed with bright yellow flowers through genetic engineering approaches. By investigating the natural biosynthetic pathways of *Torenia hybrida*, belonging to the Scrophulariaceae family, Tanaka and co-workers demonstrated that the *A. majus* aureusidin synthase (AmAS1) is the key enzyme catalyzing aurone biosynthesis from chalcones. However, to accomplish aurone biosynthesis and produce yellow-colored flowers in vivo, AmAS1 must work in tandem with chalcone 4′-*O*-glucosyltransferase (4′CGT). Actually, the co-expression of both the *AmAS1* and the *4*′*CGT* genes turned out to be sufficient for the accumulation of aureusidin-6-*O*-glucoside in the flowers of transgenic plants, thus opening up the obtainment of novel bright-yellow flowers for plant species lacking this color variant [16].

## 3. Aurones as Functional Agents 

By virtue of their poly-hydroxylated nature, aurones are able to quench reactive oxygen species (ROS), thus showing antioxidant properties. In analogy with the higher homologous flavones, the main mechanism by which aurones exert their protective role is by transferring an H atom to ROS, thus becoming radical species [17]. In addition, they may also transfer a single electron, giving rise to a radical cation. The H atoms of the hydroxy groups in both positions 3′ and 4′ of the benzylidene residue are considered to be the first shield to the attack of ROS. Once formed, the phenoxy radical may be easily stabilized thanks to the assistance of the carbon atoms in positions 2 and 3 of the nucleus, and also by the exocyclic oxygen atom in position 3, thus allowing the radical to be less prone to act as a pro-oxidant [18]. Accordingly, a high potential for ROS scavenger activity has been acknowledged for all the aurones showing a 4′-hydroxy-, as well as a 3′,4′-dihydroxy- and also a 3′,4′,5′-trihydroxy- substitution patterns. Thanks to a quantum chemical investigation, Senthil Kumar and co-workers demonstrated the high antioxidant properties of bracteatin (**7**, Figure 2), whose poly-substitution pattern on the pendant benzylidene residue allows the lowest energy for both the H-atom and electron transfer mechanism [19].

**Figure 3:**
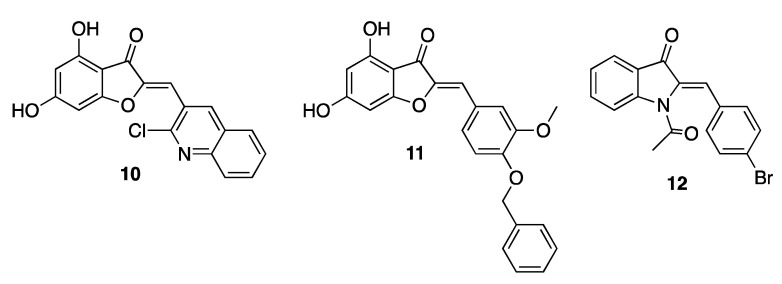
Representative synthetic antibacterial aurones.

Aurones have proved to be effective against *Escherichia coli*, *Bacillus subtilis*, *Staphylococcus aureus*, *Klebsiella pneumoniae*, *Proteus vulgaris*, and *Mycobacterium tuberculosis*, thus showing a broad-spectrum antibacterial activity. In addition to natural derivatives, a number of synthetic aurones were also developed as active agents, highlighting the importance of a diversified 2-substitution pattern to achieve a relevant efficacy. In particular, enlargement of the aromatic area in this position, as in derivatives **10** and **11** (Figure 3), provided the most performing compounds, showing minimal inhibitory concentrations (MICs) in the micromolar/submicromolar range against several pathogen bacteria. Similar functional results were also achieved when the benzofuranone core was replaced with the bioisosteric indolone, as in derivative **12** (Figure 3) [20,21,22].

**Figure 4:**
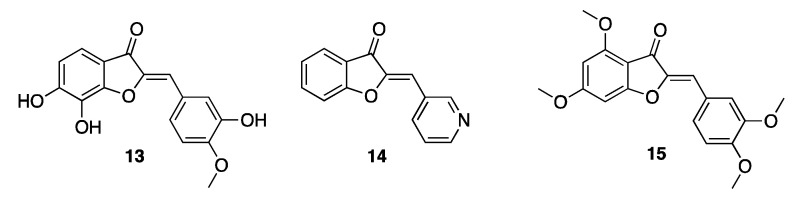
Representative natural and synthetic antifungal aurones.

As for the antifungal properties, aurones are mainly effective against *Aspergillus fumigatus*, *Aspergillus niger*, *Trichoderma virdie*, and *Penicillium chrysogenum*. Investigations into *Candida albicans* have demonstrated once more the importance of the 2-arylidene moiety for the antifungal activity. Derivatives **13** and **14** counteracted *Candida* spp., also displaying anti-biofilm activity for mid-maturation growth [23]. Aurones demonstrated insecticidal activity when tested against the larvae of *Spodoptera litura* [24]. In this case, the best activity was obtained in the presence of methoxylated derivatives. In particular, the 3′,4,4′,6 tetramethoxy-aurone **15** (Figure 4) is the compound that displayed the strongest insecticidal activity among the tested ones, thus highlighting positions 4, 6, 3′, and 6′ of the nucleus as those worth substituting with a view to implementing the activity. On the contrary, when the methoxy substituents were inserted in position 5 or 5′ the activity proved to decrease. Interestingly, the 3′,4,4′,6 tetramethoxy-aurone is also one of the main constituents isolated from the extract of *Cyperus radians*, an example of the Cyperaceae family that produce poly-methylated aurones as part of the body’s chemical defense system.

The anti-malarian activity of a number of aurone derivatives has been demonstrated by several authors, who investigated both natural derivatives and synthetic analogues [25,26,27]. Analyses of structure–activity relationships have highlighted the importance of the substitution patterns in positions 4 and 6 of the benzofurane ring. In particular, the best in vitro anti-malarian efficacy was obtained in the presence of a halogen atom in position 4 of the nucleus and/or an amino group in position 6. Significantly, all the compounds turned out to be non-cytotoxic when tested in human cells. However, when the best performing derivative was tested in vivo in laboratory animals stricken by Plasmodium falciparum, the results were rather disappointing. Actually, as often occurs in the case of natural poly-hydroxylated derivatives, the functional efficacy observed in in vitro tests is hardly replicated in in vivo models, due to bioavailability limits that characterize this kind of compound.

Many aurones have also been evaluated for their inhibitory activity against the glycoprotein neuraminidase, which is involved in the infection processes of the most common influenza viruses. In this case, the analysis of the structure–activity relationship showed that the key structural elements of the natural compounds, represented by a hydroxyl group in position 4 or 6 of the nucleus and a double bond in position 2, are essential for the activity [28].

**Figure 5:**
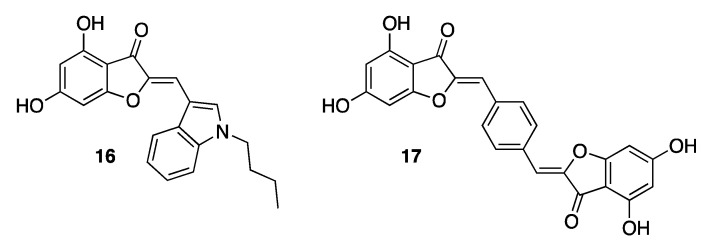
Representative examples of synthetic anti-HCV aurones.

In 2011, some aurones were identified as inhibitors of the RNA polymerase RdRp, exploited by hepatitis C virus (HCV) for replication. Due to the extreme heterogeneity of the viral genome of the causative agent of hepatitis C, to date it has not yet been possible to develop a vaccine. Indeed, eight genotypes of HCV are currently known, each differing by 30% in nucleotide sequence [29]. For many years, the weak points of HCV have been found to make the infection, if not inert, at least less dangerous. In this regard, the research community has recently been focusing on RdRp, which is a key enzyme for viral replication [30]. Nucleoside and nucleotide analogues have been shown to target the active site of RdRp, but also non-nucleoside derivatives have been disclosed as allosteric inhibitors. Regarding aurones, aureusidin **6** proved to inhibit RdRp potently, showing an IC_50_ value of 5.2 μM. Thanks to the use of a classical medicinal chemistry approach, it has been possible to identify both substitutes and ring positions conferring to aurones the best inhibitory potency. The presence of hydroxy groups in positions 4 and 6 of the nucleus turned out to be crucial for the activity. On the contrary, replacement of the hydroxy groups with methoxy substituents in the same positions resulted in a loss of activity. Different aromatic substituents were also investigated in position 2 of the benzofuran ring, demonstrating that the insertion of an indole nucleus in this position, as in compound **16** (IC_50_ 2.2 μM, Figure 5), significantly ameliorated the antiviral activity of the natural **6** [31]. Remarkable efficacy was also seen in the case of pseudodimeric compounds such as derivative **17** (IC_50_ 1.3 μM, Figure 5) [32]. Aurones displaying the most promising RdRp inhibitory activity did not show any cytotoxic effect on human cells, thus showing that they are prominent candidates for the obtainment of anti-HCV agents.

**Figure 6:**
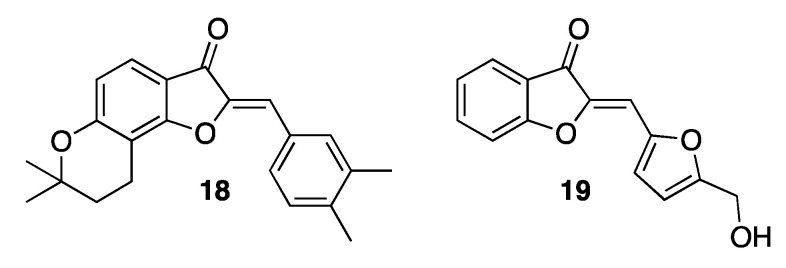
Representative examples of synthetic anti-inflammatory aurones.

Aurones and their derivatives have also been described as effective anti-inflammatory agents [6]. Actually, they are reported to inhibit the production of key cytokines such as TNF-α (tumor necrosis factor-alpha) and IL-6 (interleukin-6), released in most inflammatory processes and involved in many diseases such as autoimmune ones, diabetes, atherosclerosis, and cancer. Sulfuretin counteracts the activities of nitric oxide (NO) and prostaglandin E_2_ (PGE2), both pro-inflammatory molecules [32]. The presence of a hydroxy group in position 6 of the nucleus boosts the inhibition of PGE_2_ production, while its replacement with a methoxy group potentiates the inhibition of NO production. In any case, both substituents are crucial to combine the highest anti-inflammatory activity with the lowest toxicity. A number of sulfuretin derivatives have also been described, produced by modifying either the benzo-fused ring or the 2-benzylidene residue, or even both, which proved to be better in fighting inflammation. For example, the insertion of the 6-hydroxy substituent into a dihydropyran residue, as in compound **18** (Figure 6) [33], or the replacement of the 2-benzylidene residue with an heteroaryl moiety, as in compound **19** (Figure 6) [34].

**Figure 7:**
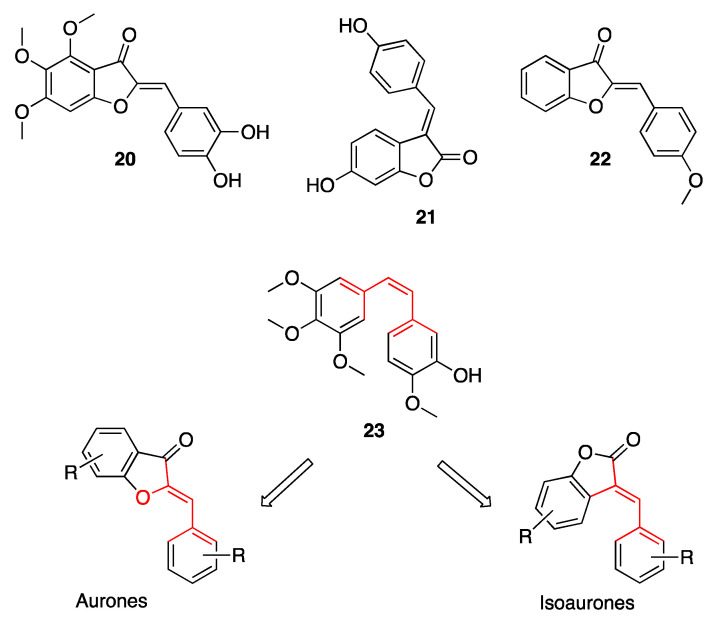
Representative examples of aurones endowed with anti-tumor efficacy.

However, most of the functional studies described by the literature aim at demonstrating the anti-cancer potential of aurones. Chemotherapy has always been the most powerful weapon at our disposal in the fight against cancer. Nevertheless, due to its high toxicity and the severe implications of resistance to the known anti-cancer drugs, the development of *magic bullets* for molecular targeted therapy has been progressing more and more. This is where aurones and their potential as anti-cancer agents come into play. The versatility of these natural compounds lies in the simplicity of their structure, which enables them to interact with key enzymes involved in tumor development. The first ever recorded antitumor activity of aurones has been described by Huang and co-workers, who demonstrated the ability of hamiltrone **20** (Figure 7), the 4,5,6-trimethoxy-substituted aurone obtained from the shrub *Uvaria hamiltonii*, to modulate the scissoring activity against the double-stranded DNA, thus damaging the DNA of proliferating cells [35]. From then on, a number of scientific reports have been published, thoroughly reviewed by Alsayari and co-workers, testifying to the ability of both aurones and their regioisomes isoaurones to interact with several key cancer targets.

Suzuki and co-workers demonstrated the anti-tumor efficacy of isoaurostatin **21** (Figure 7), the fungal metabolite of *Thermomonospora alba*, as the result of its inhibitory activity against Topoisomerase I [36], while Priyadarshani and co-workers reported on the ability of (*Z*)-2-(4-methoxybenzylidene)benzofuran-3(2*H*)-one **22** (Figure 7) to inhibit Topoisomerase II [37]. Overall, the benzofuran-3(2*H*)-one scaffold gave evidence these key enzymes regulating DNA replication and transcription, once decorated with appropriate substituents on the pendant phenyl ring. In any case, the reference pharmacophoric structure of both aurones and isoaurones, characterized by two main aromatic areas connected by a double bond having Z geometry, has been deemed by several authors to overlap the one of combrestatin A-4 **23** (Figure 7), a well-known tubulin polymerization inhibitor. This would explain the anti-cancer activity shown by several natural and synthetic aurones, which proved to interact at the colchicine binding site of tubulin, thus arresting cell cycle at the G2/M level.

Both aurones and isoaurones, as well as the synthetic benzofuranone derivatives closely related to them, have also been reported to interact with additional anti-tumor targets. These include the serine/threonine cyclin-dependent kinases CDK 1 and 2, playing a key role in cell cycle regulation [38], the Hypoxia-inducible Factor-1 (HIF-1), a marker reflecting angiogenesic activity in cancer cells [39,40], but also protein kinases such as fibroblast growth factor receptor, FGFR, which is involved in cancer development and progression [41], and sphingosine kinase, whose overexpression has been associated with tumor angiogenesis and resistance to radiation and chemotherapy [42]. Examples of synthetic aurones have also been described for their ability to dissipate the hyperpolarization of mitochondrial membranes of cancer cells, arresting cell cycle [43]. Instead, contrasting results were obtained against histone deacetylases (HDACs), a family of enzymes that, once mutated or overexpressed, induce the aberrant expression of genes involved in cell proliferation and apoptosis. Indeed, while Zwick and co-workers claimed an HDAC inhibitory activity in the micromolar range for a number of poly-hydroxylated aurones [44], Itoh and co-workers warned against the activity displayed by those aurones bearing a catechol fragment on the 2-benzylidene residue [45]. Actually, according to the authors, this structural motif proved to interfere with the proper functioning of the in vitro assay components, thus causing false positives.

The development of the ideal anti-tumor agent cannot ignore the multi-drug resistance that cancer cells are able to put in place. The activation of repairing and detoxifying systems, as well as a reduced uptake of drugs due to cell adhesion barriers, are just a few examples of strategies in force to malignancies to protect themselves against the toxic effects of drugs. However, the most effective resistance mechanism is mainly due to the activity of the ATP-dependent efflux pumps of the ABC family, such as the P-glycoprotein (P-gp/ABCB1), multidrug resistance-associated protein 2 (MRP2/ABCC2), and the breast cancer resistance protein (BCRP/ABCG2), whose overexpression limits the prolonged and effective activity of chemotherapeutic drugs [46]. Additionally, in this case, aurones come in handy to obtain effective ATP-binding cassette inhibitors. Moreover, by suitably modifying the substitution pattern on either the benzylidene moiety or the benzo-fused ring, a selectivity profile against the three different efflux pumps may be also obtained. Indeed, several halogenated aurones have been described as effective P-gp inhibitors able to increase daunorubicin cyctotoxicity when tested in vitro in the chronic myelocytic leukemia K562 cell line [47] and paclitaxel accumulation in the human breast MDA-MB-436 cell line [48]. Instead, when the benzylidene moiety was replaced with an indolylmethylene residue, the resulting compound turned out to be effective against the ABCC2 protein [49]. Poly-methoxylated derivatives proved to interact with the ABCG2 efflux pump, inducing a mitoxantrone accumulation in cell lines [50]. All things considered, suitably modified members of this class of natural compounds offer the opportunity to sum up the ability to interact with key molecular anti-cancer targets with the potential to modulate the activity of efflux pumps, thus embodying the ideal anti-cancer agents. 

**Figure 8:**
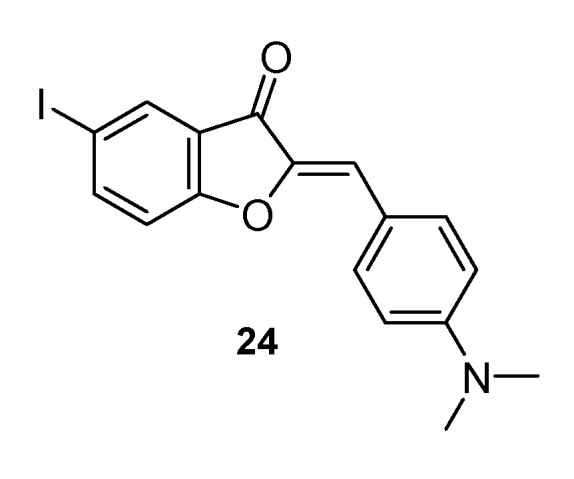
Synthetic aurone endowed with staining property.

Finally, it is worth mentioning the fluorescent potentials of this class of natural compounds. Organic molecules having fluorescence properties in the visible region of the electromagnetic spectrum are very useful investigative tools in biological systems. However, to be used for this purpose, compounds should bring only minimal perturbations to the biological macromolecules under study, to highlight their characteristics as accurately as possible to the physiological situation. Therefore, they should be characterized by rather small dimensions. Unfortunately, the currently available fluorophores, including xanthenes such as fluorescein and eosin, BODIPY, and cyanines, do not fully comply with this criterium. Shanker and co-workers demonstrated the fluorescent potential of some aurone derivatives, suggesting their possible use for biomolecular investigations [51]. Significantly, even the largest example proposed by the authors is smaller than xanthene dyes, and this structural characteristic is particularly advantageous for the use of the compounds. Studies on the potential application of aurone derivatives in the field of fluorescence are still ongoing but are proving to be rather promising. Ono and co-workers reported the ability of a synthetic aurone, 2-[(4-dimethylaminophenyl)methylene]-5-iodo-3(2*H*)-benzofuranone **24** (Figure 8), to efficiently stain Alzheimer’s mouse brain sections, thanks to the high binding affinity to the peptides of the Aβ aggregates displayed by the compound. A concomitant good brain penetration and fast washout, demonstrated through biodistribution studies carried out on normal mice, make the compound the ideal prototype probe for detecting amyloid plaques in the brain of people affected by Alzheimer disease, thus opening up a further role for these compounds [52].

The remarkable versatility of aurones, evidenced by an increasing amount of scientific evidence, makes these compounds worthy of consideration by the scientific community. However, the prospect of using them for both technological and functional purposes raises the problem of their availability. This is why, in addition to further clarifying their biological significance, it is crucial to develop synthetic strategies to obtain them in high amounts.

## 4. Synthetic Strategies to Obtain Aurones

A number of synthetic approaches have been proposed throughout years, to both replicate in the laboratory the natural aurones and obtain novel analogues. Attempts made by different researchers aimed at rebuilding the 2-functionalized benzofurane scaffold following different chemical strategies, all reviewed hereinafter.

### 4.1. Functionalization of the Benzofurane Nucleus

**Scheme 1:**
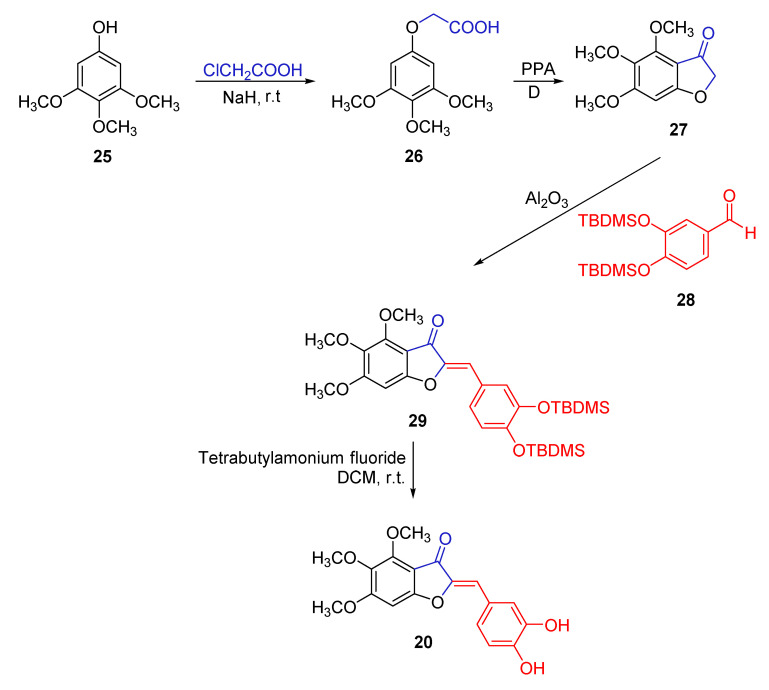
Synthesis of hamiltrone according to Lawrence and co-workers [53].

Lawrence and co-workers investigated the natural biosynthesis of hamiltrone **20**, the aurone isolated from the *U. hamiltonii*, and putted in place a synthetic strategy that, in addition to the natural compound, allowed to get to a series of structural analogues [53]. As for hamiltrone, starting from the 3,4,5-trimethoxyphenol derivative **25**, the authors obtained the corresponding phenoxyacetic acid **26**, by reaction with chloroacetic acid, then cyclized the acid to the key intermediate benzofuranone **27**, by treatment with polyphosphoric acid, achieving a 51% yield of the target heterocycle. The reaction of **27** with the TBDMS-protected benzaldehyde **28**, in the presence of neutral alumina, allowed to obtain the protected aurone **29**, which lastly afforded the target hamiltrone **20** by reaction with tetra-butylammonium fluoride. The proposed synthetic pathway proved to work well using also differently substituted benzaldehydes, carrying either electron-withdrawing atoms, such as halogens, or electron-donating groups, such as the methoxy one. Altogether, the followed pathway only resulted in the (*Z*)-isomer, being more thermodynamically stable than the (*E*)-counterpart, as reported by the authors (Scheme 1).

**Scheme 2:**
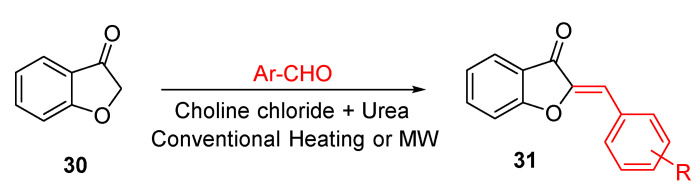
Synthesis of (*Z*)-2-benzylidenebenzofuran-3(2*H*)-ones according to Hawkins and co-workers [54] and Taylor and co-workers [55].

A similar functionalization of the benzofurane ring was also carried out in 2013 by Hawkins and co-workers, who exploited a mixture of choline chloride and urea as the catalytic deep eutectic solvent to insert the benzylidene residue in position 2 of the benzofurane core **30** (Scheme 2) [54]. By heating at 80 °C equimolar amount of benzofurane and a suitably substituted arylaldehyde in a sealed vial, in the presence of the deep eutectic solvent, aurone derivatives were obtained in 40–78% yields within 12–48 h. The reaction proceeds under relatively mild conditions, without the need for basic and acid catalysts, thus proving to be invaluable for sensitive reactants. A few years later, Taylor and co-workers improved the procedure by pairing the use of the deep eutectic solvent with the highly performing microwave irradiation, thus maximizing both the yield of the obtained aurones and the time of reactions. Moreover, compounds not achievable with conventional heating were also obtained (Scheme 2) [55]. 

**Scheme 3:**
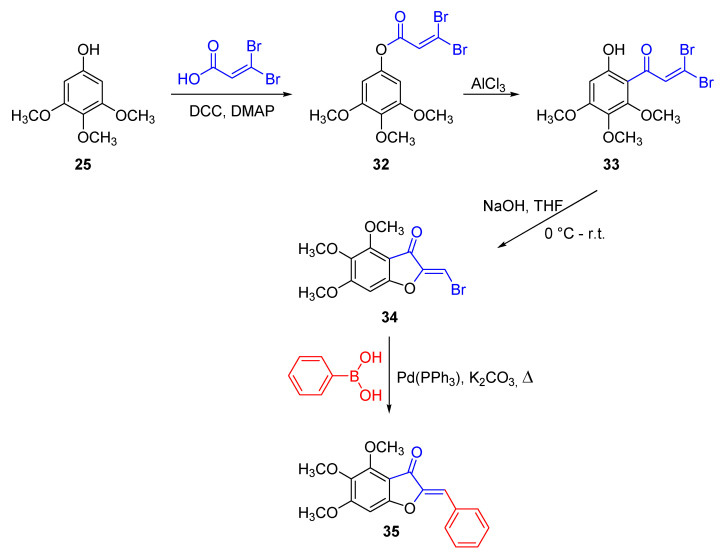
Synthesis of 4,5,6-trimethoxyaurone according to Kraus and Gupta [57].

Kraus and Gupta took advantage of a Steglich esterification [56], carried out in the presence of dicyclohexylcarbodiimide as the coupling reagent and 4-dimethylaminopiridine as the catalyst, to turn the phenol derivative **25** into the 3,4,5-trimethoxyphenyl-3,3-dibromoacrylate **32**, by reaction with 3,3-dibromoacrylic acid. A Fries rearrangement of the ester **32** into the ketone **33**, followed by its cyclization in dilute sodium hydroxide and THF, afforded the 2-bromomethylene benzofuran-3(2*H*)-one **34**. A conventional Suzuky–Miyaura coupling finally led to the aurone **35** (Scheme 3) [57].

**Scheme 4:**
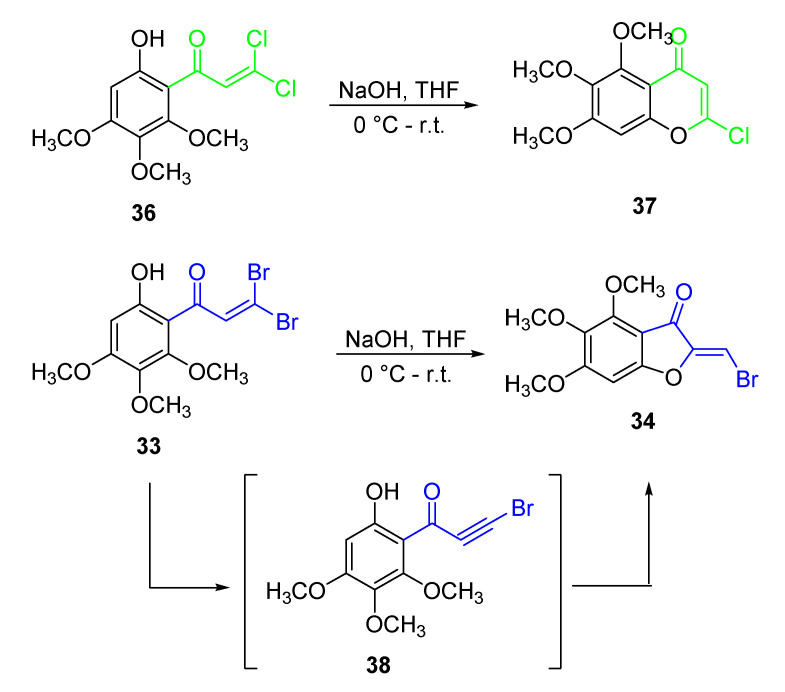
Reactivity of α-dihalo-prop-2-en-1-ones in comparison.

Intriguingly, the cyclization of the 3,3-dibromo-1-(substituted)prop-2-en-1-one **33** into the 6:5 aurone **34** turned out to be a surprising occurrence. Actually, the authors expected to obtain the higher homologous 6:6 chromone as, using the same procedure in the presence of 3,3-dichloroacrylic acid as the starting reactant, and treating the key intermediate **36** with dilute base, the corresponding 2-chloro-5,6,7-trimethoxy-4*H*-chromen-4-one **37** was obtained (Scheme 4).

However, in the case of the α-dibromo derivative **33**, two different and competing reaction may be assumed. Indeed, the cyclization reaction driven by the phenolic oxygen, and leading to the 6:6 nucleus, has to deal with the dehydrobromination reaction affording the bromo-acetylenic intermediate **38**, which in turn can cyclize to the 6:5 heterocycle. If the elimination reaction prevails, the aurone proves to be the predominant product (Scheme 4).

### 4.2. Cyclization of Chalcones

**Scheme 5:**
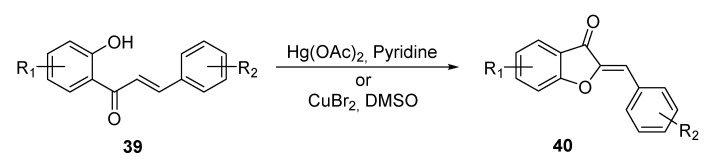
Cyclization of chalcones according to Agrawal and Soni [58].

Agrawal and Soni focused on 2-hydroxycalcones **39**, achieving their cyclization to aurones **40** in oxidant conditions by means of Hg(OAc)_2_ in the presence of pyridine. CuBr_2_ in DMSO was also used to induce alternative cyclization conditions, leading to aurones in comparable yields [58] (Scheme 5). In both cases, the oxidant mixture promotes a *5-exo-trig* cyclization to the 6:5 nucleus deterring the also possible *6-endo-trig* cyclization to the 6:6 higher homologous. According to the authors, coordination between the oxygen atom of the hydroxy group and the metal species occurs, which prevents the six-member cyclization.

**Scheme 6:**
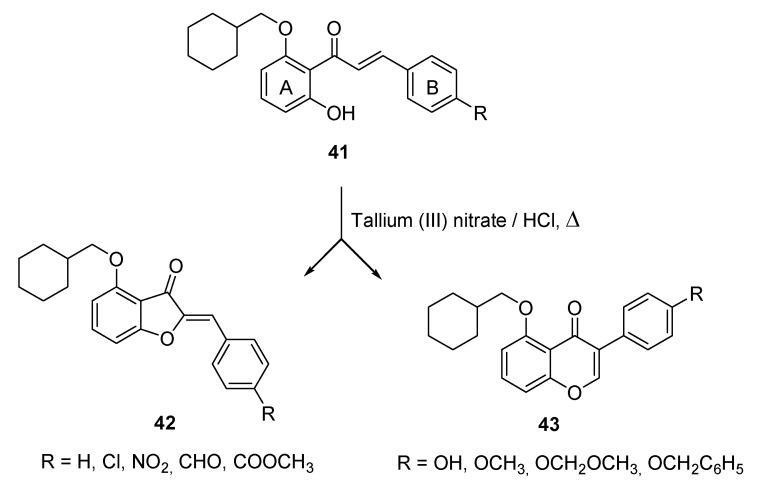
Cyclization of chalcones according to Thanigaimalai and Yang [59].

Additionally, Thanigaimalai and Yang pursued the cyclization of chalcones by exploiting thallium (III) nitrate in methanol solution followed by the addition of hydrochloric acid as the oxidant condition [59]. Using 1-(2-(cyclohexylmethoxy)-6-hydroxyphenyl)-3-phenylprop-2-en-1-one **41** as the starting material, they obtained either the aurone derivatives **42** or the isoflavone derivatives **43** depending on the substitution pattern on the B ring of the chalcone. In particular, the presence of electron-withdrawing substituents such as chloro, nitro, formyl, and methoxycarbonyl in the *para* position of the phenyl ring was mandatory to produce aurones, while their replacement with strong electron-donating groups such as hydroxy, methoxy, methoxymethoxy, and benzyloxy led to the obtainment of isoflavones. The presence of the weak electron-donating ethyl group allowed to obtain a 1:1 mixture of both isoflavones and aurones, while the presence of a carboxylic residue left the chalcone unreacted (Scheme 6).

**Scheme 7:**
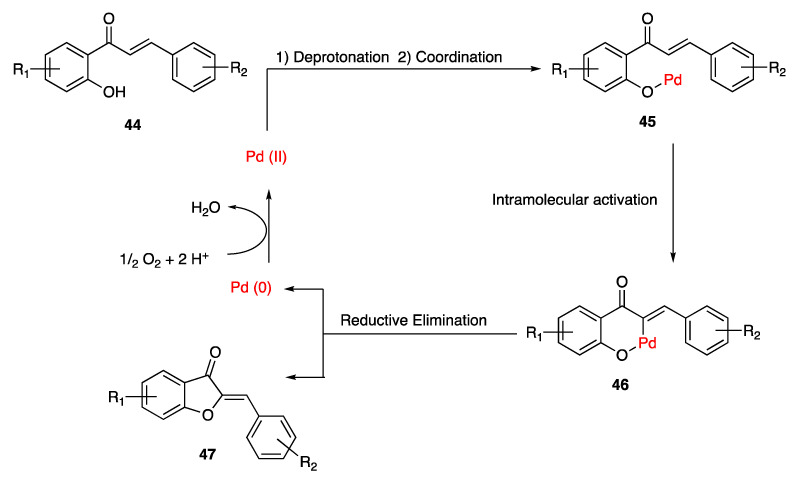
Cyclization of chalcones according to Yatabe and co-workers [60].

A further example of chalcones cyclization has been provided by Yatabe and co-workers, who produced the expected aurones by treating the starting compounds with oxygen from air in the presence of an heterogeneous catalyst, made of Pd-on-Au nanoparticles supported on CeO_2_ [60]. At first, the deprotonation of the hydroxy group of **44** occurs, which allows the coordination of chalcone with the heterogenous Pd catalyst as in **45**. Then, a six-membered palladacycle **46** is obtained, thanks to an intramolecular olefinic α-C-H activation promoted by the phenoxide residue. The following reductive elimination of Pd from the palladacycle leads to the desired aurone **47** in its (Z)-predominant isoform, while the resulting reduced Pd(0) is reoxidized by oxygen from air, thus maintaining the catalytic efficiency of the system (Scheme 7).

### 4.3. Rearrangement of Six-Membered Cycles

**Scheme 8:**
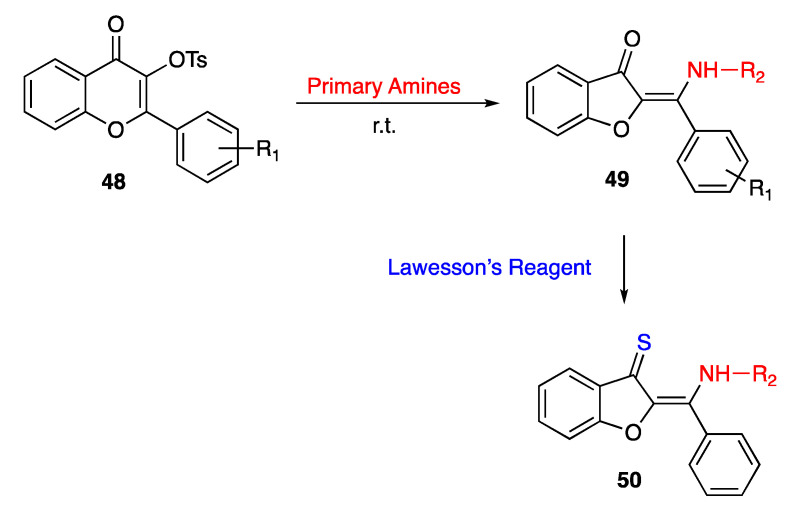
Synthesis of thioaurones according to Kandioller and co-workers [61].

In 2015, Kandioller and co-workers succeeded in turning 3-tosilflavones **48** into five-membered benzofuranes **49**, by treating the six-membered heterocycles with suitably substituted alkylamines under mild reaction conditions. The rearrangement reactions proved to produce a high yield, but the resulting products were obtained as an *E*-*Z* mixture [61]. However, when the mixed isomers were treated with Lawesson’s reagent, the thermodynamically favored *E*-isomers were isolated as the only 3(2*H*)-thioaurones **50** [61] (Scheme 8). 

**Scheme 9:**
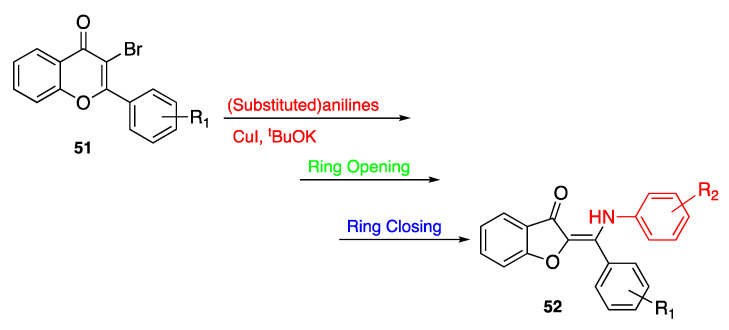
Rearrangement of flavones according to Parveen and Ahmed [62].

A few years later, Parveen and Ahmed yielded similar rearrangement results by using 3-bromoflavones **51** as the starting compounds [62]. Actually, by treatment with aniline derivatives in the presence of *t*-Bu-OK and CuI as the catalysts, the six-membered derivatives underwent a three-step cascade reaction including an Aza-Michael addition followed by ring opening and ring closing, leading to 2-aminated aurones **52** in their *trans*-conformation. The rearrangement took place under mild reaction conditions, proving to be effective in the presence of differently substituted aniline derivatives as well as N-phenylurea, but turned out to be unattainable when benzamide, 2-aminopyridine, and also piperidine were used as the key nucleophiles (Scheme 9). 

As a whole, the rearrangement of flavones in the presence of nitrogen-based nucleophiles provides the insertion of an additional substituent in position 2 of the heterocyclic core, thus allowing to expand the structural variability of the benzofuran-3(2*H*)-ones and, accordingly, their usability in the most varied fields.

### 4.4. Reactions of Annulation

A number of annulation reactions has been proposed to obtain aurones, all starting from suitably substituted *ortho*-halo phenols. The carbonylative cyclization was carried out by using different types of catalysts, generally applying mild reaction conditions. In some cases, the target products were obtained as a mixture with the upper homologous flavones, thus forcing a further separation passage to obtain the pure aurones. Among the available catalysts, those made from palladium turned out to be the most effective ones, being used in most of the synthetic procedures described in the literature.

**Scheme 10:**
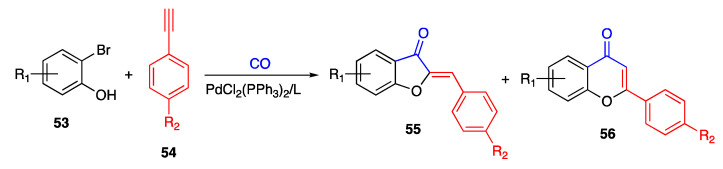
Annulation of 2-bromo-phenols according to Liu and co-workers [63].

In 2013, Liu and co-workers described a PdCl_2_(PPhe_3_)_2_–benzimidazolium complex as a viable catalyst to produce aurones by reacting suitably substituted *ortho*-bromo-phenols **53** and ethynylbenzenes **54** [63]. The target compounds **55** were obtained under mild conditions, although in a mixture with 20–40% of the parent flavones **56** (Scheme 10).

**Scheme 11:**
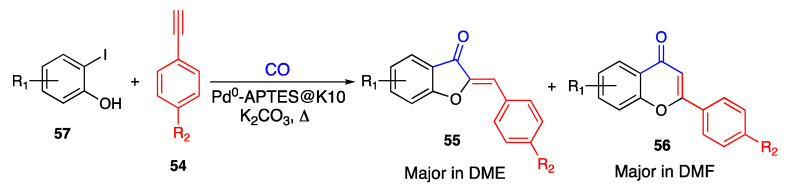
Annulation of 2-iodo-phenols according to Chavan and co-workers [64].

A similar reaction was described by Chavan and co-workers, who used Pd^0^ complexed with 3-aminopropyl-triethoxysilane (APTES@K10) as the catalyst [64]. Additionally, in this case, the reaction of *ortho*-iodo-phenols **57** with terminal alkynes **54** resulted in the target 6:5 aurones **55**, which were eventually isolated with the upper 6:6 counterpart flavones **56**. However, the authors succeeded in ensuring the obtainment of a heterocycle rather than the other one by using different solvents. Actually, in the presence of 1,2-dimethoxyethane (DME), the regioselective 5-exo cyclization was predominant, leading to the desirable aurones as the reference reaction products. Instead, the 6-endo cyclization mainly took place when using DMF, thus producing the parent flavones (Scheme 11).

**Scheme 12:**
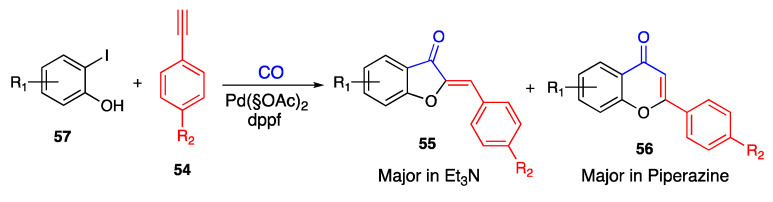
Annulation of 2-iodo-phenols according to Xu and co-workers [65].

An analogous example of solvent-driven regioselective carbonylative cyclization was given by Xu and co-workers, who obtained the 6:5 membered heterocycle and the 6:6 counterpart according to the solvent used [65]. In particular, triethylamine mainly led to the target aurones, managing a 5-exo cyclization, while piperazine preferentially pushed a 6-endo reaction, thus allowing us to obtain the corresponding flavones (Scheme 12).

**Scheme 13:**
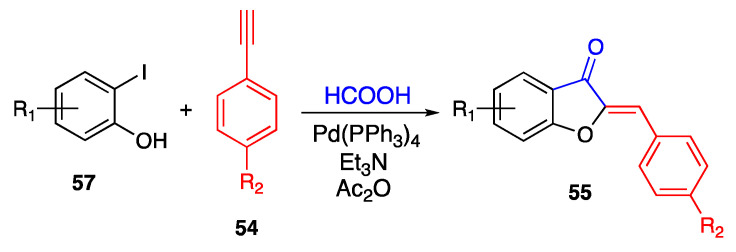
Annulation of 2-iodo-phenols according to Qi and co-workers [66].

However, when formic acid was used as the CO source, the annulation reaction proceeded to the target aurones as the reference products. Indeed, Qi and co-workers described the obtainment of differently substituted aurones **55** by reaction between *ortho*-iodo-phenols **57** and terminal alkynes **54**, carried out in the presence of tetrakis(triphenylphosphine)palladium as the catalyst [66] (Scheme 13).

**Scheme 14:**
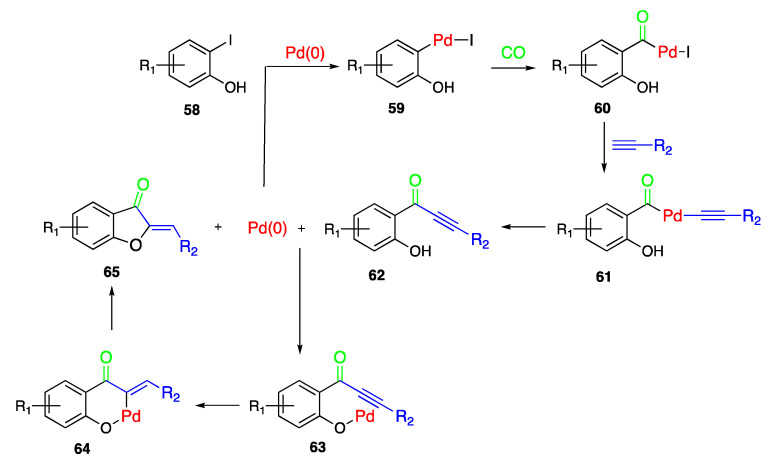
Reaction mechanism proposed for the Pd-catalyzed annulation.

Regarding the Pd-catalyzed annulation reactions, a common mechanism may be assumed providing for the insertion of the carbonyl residue as the first step and the addition of the benzylidene moiety as the second one, both of them assisted by the metal catalyst. In particular, the *ortho*-iodo phenol **58** may be subjected to an oxidative addition of Pd(0), thus being converted into the arylpalladium derivative **59**, and this latter turns into the aroylpalladium derivative **60** due to the insertion of CO. The following sequentially alkyne attack and Pd(0) elimination give the key aryl-alkyn-one intermediate **62**, which is then complexed by Pd(0), to give **63**, cyclized to the palladacycle **64**, and finally transformed into the expected aurone **65** (Scheme 14).

Although most researchers have focused on palladium-based compounds as the key catalysts to produce the target aurones, few examples have also been reported demonstrating the usefulness of additional metals such as rhodium, copper, and cesium.

**Scheme 15:**
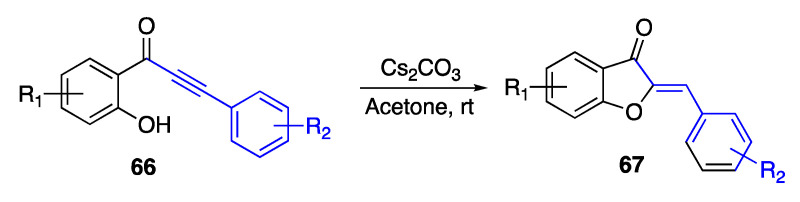
Annulation of 2-alkynoylphenols according to Taylor and Bolshan [67].

Starting from *ortho*-alkynoylphenols **47**, Taylor and Bolshan obtained a series of aurones **44** by using cesium carbonate in acetone solution [67]. The adopted experimental conditions promoted an intramolecular 5-exo cyclization as the main reaction, thus leading to the benzofurane derivatives in good to high yields (Scheme 15).

**Scheme 16:**
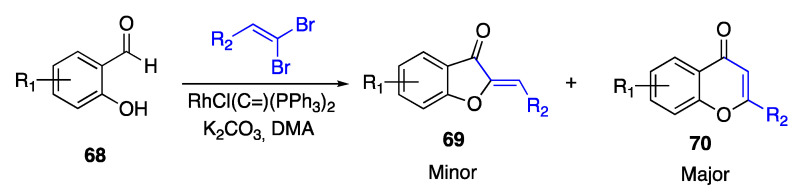
Annulation of salicylaldehydes according to Rao and Ramakrishna [68].

A rhodium-based catalyst was used in basic medium by Rao and Ramakrishna to cyclize 1-bromo-alkynes, obtained in turn by the alkynylation of suitably substituted salicylaldehydes and 1,1-dibromoalkenes [68]. However, differently from the previously described strategy, the synthetic procedure adopted by Rao and Ramakrishna led mainly to the synthesis of the 6:6 heterocyles, with the 6:5 aurones being obtained in minor yields (Scheme 16).

## 5. Conclusions

Although having mentioned by the scientific literature since the beginning of the twentieth century, aurones have been long neglected by researchers to the benefit of the more homologous flavonoids. However, they are recently regaining interest, as their functional properties are unveiled. Being key determinants for color plants, these compounds have an undoubted technological role as natural dyes. At the same time, being acknowledged for their antioxidant, anti-inflammatory, antibacterial, antiviral, antimalarial, and anticancer properties, they may be claimed to be innovative drug candidates. Moreover, they may also be considered as viable diagnostic tools, due to their fluorescent properties. In addition to naturally occurring aurones, an increasing number of synthetic analogues have also been described, being similar though their improved functional properties, which further expands this class of compounds. The amount of scientific data collected so far clearly demonstrate the potential for the development of these natural derivatives and their synthetic analogues, yet we are only beginning to comprehend their real usability. Actually, a number of crucial matters still need to be clarified. The synthetic supply of the compounds provides an example: according to the literature, a straightforward, cost-effective, and environmentally friendly chemical procedure to achieve benzofuran derivatives on an industrial scale has yet to be finalized. Then, and more importantly, it is necessary to tidy up the information on both the biological activity and the bioavailability of these compounds. Indeed, as is often the case with natural products, aurones have a multi-functional profile. Although sometimes it may beneficial, depending on the disease in question, a redundancy of interactions with different targets often precludes the development of a compound as a real drug. Moreover, being characterized by polyphenolic structures, these derivatives are generally marked by unfavorable pharmacokinetic properties. Therefore, it is highly desirable to deepen the interactions of aurones with the identified molecular targets and the novel forthcoming ones, by using crystallographic and computations studies, to highlight the key structural discriminants for activity. In addition, a mathematical characterization of the time course of compounds’ absorption, distribution, metabolism, and excretion is also necessary. These investigations will allow us to obtain compounds endowed with a clearly defined drug-like profile.

All things considered, even though it has already proven to be promising, research on aurones is just getting started.

## Data Availability

Not applicable.
